# Supplementary material for: Unmet Needs Mediate the Impact of Fear of Cancer Recurrence on Screening Participation Among Cancer Survivors: A Cross-Sectional Study
Source: Healthcare (Basel). 2025 May 19;13(10):1184. doi: 10.3390/healthcare13101184 (PMC12111079; doi:10.3390/healthcare13101184)
Supplement: Supplementary file 1 [file healthcare-13-01184-s001.zip › healthcare-3614639-supplementary.pdf]

Table S1. Distribution of unmet needs based on selected domains of the Cancer Patient Needs Assessment Tool.

	**No need/met need** *N* **(%)**	**Unmet need, weak** *N* **(%)**	**Unmet need, moderate** *N* **(%)**	**Unmet need, strong** *N* **(%)**	**Unmet need, m oderate-strong** *N* **(%)**
Information and education					
I needed information about my current condition and prognosis.	31 (9.5)	123 (37.7)	90 (27.6)	82 (25.2)	172 (52.8)
I needed information on tests and treatments.	184 (56.4)	60 (18.4)	50 (15.3)	32 (9.8)	82 (25.1)
I needed to know which symptoms required medical attention.	35 (10.7)	111 (34.0)	97 (29.8)	83 (25.5)	180 (55.3)
I needed clear and accurate explanations of medications.	26 (8.0)	110 (33.7)	89 (27.3)	101 (31.0)	190 (58.3)
I needed guidance on self-care at home (e.g., exercise).	107 (32.8)	71 (21.8)	75 (23.0)	73 (22.4)	148 (45.4)
I needed information on complementary and alternative therapies.	51 (15.6)	90 (27.6)	95 (29.1)	90 (27.6)	185 (56.7)
I needed dietary advice (e.g., recommended and avoided foods).	92 (28.2)	91 (27.9)	84 (25.8)	59 (18.1)	143 (43.9)
I needed information about treatment centers and physicians.	29 (8.9)	93 (28.5)	92 (28.2)	112 (34.4)	204 (62.6)
I needed information on financial support for cancer patients.	65 (19.9)	95 (29.1)	83 (25.5)	83 (25.5)	166 (51.0)
I needed information about hospice and palliative care.	33 (10.1)	65 (19.9)	83 (25.5)	145 (44.5)	228 (70.0)
Psychological issues					
I needed help managing general anxiety.	85 (26.1)	113 (34.7)	75 (23.0)	53 (16.3)	128 (39.3)
I needed help coping with fear of recurrence.	128 (39.3)	92 (28.2)	45 (13.8)	61 (18.7)	106 (32.5)
I needed help dealing with concerns about treatment side effects.	44 (13.5)	114 (35.0)	78 (23.9)	90 (27.6)	168 (51.5)
I needed support due to worries about my family.	51 (15.6)	113 (34.7)	74 (22.7)	88 (27.0)	162 (49.7)
I needed support due to fear of being a burden.	95 (29.1)	96 (29.4)	76 (23.3)	59 (18.1)	135 (41.4)
I needed help with depression.	98 (30.1)	100 (30.7)	77 (23.6)	51 (15.6)	128 (39.2)
I needed help managing anger or irritability.	119 (36.5)	85 (26.1)	71 (21.8)	51 (15.6)	122 (37.4)
I needed help with loneliness or feeling isolated.	109 (33.4)	96 (29.4)	62 (19.0)	59 (18.1)	121 (37.1)
I needed help adjusting to changed roles at home, work, or in so	119 (36.5)	91 (27.9)	65 (19.9)	51 (15.6)	116 (35.5)

ciety.					
I needed help accepting changes in my appearance.	98 (30.1)	106 (32.5)	62 (19.0)	60 (18.4)	122 (37.4)
Physical symptoms					
I needed help managing pain.	94 (28.8)	105 (32.2)	76 (23.3)	51 (15.6)	127 (38.9)
I needed help with fatigue.	153 (46.9)	71 (21.8)	56 (17.2)	46 (14.1)	102 (31.3)
I needed help with sleep problems.	106 (32.5)	90 (27.6)	77 (23.6)	53 (16.3)	130 (39.9)
I needed help with diarrhea or constipation.	167 (51.2)	72 (22.1)	55 (16.9)	32 (9.8)	87 (26.7)
I needed help with nausea or vomiting.	73 (22.4)	107 (32.8)	85 (26.1)	61 (18.7)	146 (44.8)
I needed help with poor appetite.	110 (33.7)	86 (26.4)	55 (16.9)	75 (23.0)	130 (39.9)
I needed help coping with hair loss.	123 (37.7)	105 (32.2)	54 (16.6)	44 (13.5)	98 (30.1)
I needed help with shortness of breath.	181 (55.5)	73 (22.4)	43 (13.2)	29 (8.9)	72 (22.1)
I needed help with numbness or body aches.	188 (57.7)	66 (20.2)	40 (12.3)	32 (9.8)	72 (22.1)
I needed help with hot flashes or flushing.	173 (53.1)	62 (19.0)	37 (11.3)	54 (16.6)	91 (27.9)
I needed help with memory or concentration problems.	169 (51.8)	69 (21.2)	57 (17.5)	31 (9.5)	88 (27.0)
I needed help with sexual health concerns.	100 (30.7)	90 (27.6)	60 (18.4)	76 (23.3)	136 (41.7)
Social support					
I wanted opportunities to share experiences (e.g., support groups).	76 (23.3)	124 (38.0)	68 (20.9)	58 (17.8)	126 (38.7)
I needed support for returning to work or finding employment.	126 (38.7)	88 (27.0)	60 (18.4)	52 (16.0)	112 (34.4)
I needed help with transportation to the hospital.	154 (47.2)	69 (21.2)	61 (18.7)	42 (12.9)	103 (31.6)
I wanted treatment closer to home.	101 (31.0)	72 (22.1)	65 (19.9)	88 (27.0)	153 (46.9)
I needed accommodation near the treatment facility.	189 (58.0)	51 (15.6)	36 (11.0)	50 (15.3)	86 (26.3)
I needed help with the financial burden of cancer.	80 (24.5)	77 (23.6)	62 (19.0)	107 (32.8)	169 (51.8)
I needed help with housekeeping or childcare.	157 (48.2)	67 (20.6)	56 (17.2)	46 (14.1)	102 (31.3)
I needed a caregiver at the hospital or at home.	185 (56.7)	69 (21.2)	41 (12.6)	31 (9.5)	72 (22.1)

Based on the Cancer Patient Needs Assessment Tool included in the Ministry of Health and Welfare 2008 report "Development of a Quality Improvement System for Cancer Care and Strategies to Enhance Cancer Coverage."
